# Warm blood cardioplegia versus cold crystalloid cardioplegia for myocardial protection during coronary artery bypass grafting surgery

**DOI:** 10.1038/s41420-018-0031-z

**Published:** 2018-02-14

**Authors:** Paolo Nardi, Calogera Pisano, Fabio Bertoldo, Sara R. Vacirca, Guglielmo Saitto, Antonino Costantino, Emanuele Bovio, Antonio Pellegrino, Giovanni Ruvolo

**Affiliations:** Cardiac Surgery Division, Tor Vergata University Hospital, Rome, Italy

## Abstract

We retrospectively analyzed early results of coronary artery bypass grafting (CABG) surgery using two different types of cardioplegia for myocardial protection: antegrade intermittent warm blood or cold crystalloid cardioplegia. From January 2015 to October 2016, 330 consecutive patients underwent isolated on-pump CABG. Cardiac arrest was obtained with use of warm blood cardioplegia (WBC group, *n* = 297) or cold crystalloid cardioplegia (CCC group, *n* = 33), according to the choice of the surgeon. Euroscore II and preoperative characteristics were similar in both groups, except for the creatinine clearance, slightly lower in WBC group (77.33 ± 27.86 mL/min versus 88.77 ± 51.02 mL/min) (*P* < 0.05). Complete revascularization was achieved in both groups. In-hospital mortality was 2.0% (*n* = 6) in WBC group, absent in CCC group. The required mean number of cardioplegia’s doses per patient was higher in WBC group (2.3 ± 0.8) versus CCC group (2.0 ± 0.7) (*P* = 0.045), despite a lower number of distal coronary artery anastomoses (2.7 ± 0.8 versus 3.2 ± 0.9) (*P* = 0.0001). Cardiopulmonary and aortic cross-clamp times were similar in both groups. The incidence of perioperative myocardial infarction (WBC group 3.4% versus CCC group 3.0%) and low cardiac output syndrome (4.4% versus 3.0%) were similar in both groups. As compared with WBC group, in CCC group CK-MB/CK ratio >10% was lower during each time points of evaluation, with a statistical significant difference at time 0 (4% ± 1.6% versus 5% ± 2.5%) (*P* = 0.021). In presence of complete revascularization, despite the value of CK-MB/CK ratio >10% was less in the CCC group, clinical results were not affected by both types of cardioplegia adopted to myocardial protection. As compared with cold crystalloid, warm blood cardioplegia requires a shorter interval of administration to achieve better myocardial protection.

## Introduction

Cardioplegia represents the most important strategy aimed to protect myocardial function during cardiac surgery and to facilitate surgical procedures providing a quiet and bloodless operative field.

Initially, cardioplegia was introduced as an agent for hypothermic hyperkaliemic arrest. Blood was then found to be an important vehicle for delivery of potassium cardioplegia^1, 2^.

Cold crystalloid cardioplegia associated with mild-to-moderate hypothermia has the advantage to decrease the oxygen consumption and offers some degree of protection during periods of low flow or low perfusion pressure. Moreover, crystalloid cardioplegia gives a better view when performing distal coronary artery anastomoses.

Warm blood cardioplegia has been proposed as a safe and reliable technique for myocardial protection, based on the rationale that blood, as opposed to crystalloid solution, can potentially improve postoperative cardiac outcomes, because it more closely approximates normal physiology, i.e., carrying oxygen to the myocardium or ensuring a less hemodilution. Nevertheless, there is still debate which is better cardioplegia for myocardial protection during cardiac surgical procedures^3–5^.

The aim of this study was to retrospectively evaluate whether in-hospital outcomes in patients undergoing coronary artery bypass grafting (CABG) surgery were different by using intermittent antegrade warm blood cardioplegia or intermittent antegrade cold crystalloid St. Thomas cardioplegia.

## Materials and methods

From January 2015 to October 2016 at the Cardiac Surgery Unit of the Tor Vergata University Hospital of Rome, 330 consecutive patients (mean age of 67 ± 9 years) underwent isolated CABG by means of cardiopulmonary bypass. Cardiac arrest was obtained using warm blood cardioplegia (warm blood cardioplegia (WBC) group, *n* = 297) or cold crystalloid St. Thomas cardioplegia (crystalloid cold cardioplegia (CCC) group, *n* = 33) on the basis of surgeons’ choice. These two groups of patients represented the object of the present study.

All patients performed preoperatively trans-thoracic echocardiography and cardiac catheterization with selective coronary angiography, and postoperatively a trans-thoracic echocardiography on the third–fourth postoperative day. Patients operated in emergency or on beating heart were excluded from the study. The study was approved by the local Institutional Review Board, which waived the need for patient consent. This study was designed to be as retrospective one.

### Data collection

In all patients were evaluated serum myocardial enzymes levels, i.e., creatine kinase (CK)-MB/CK ratio >10% and cardiac troponin I, at the end (time 0, i.e., at the admission in intensive care unit), 24 and 48 h after CABG. Perioperative myocardial infarction was defined as an increase of postoperative troponin I above10 ng/mL associated with an increase of serum CK-Muscle/Brain (MB) enzyme .10% of the total creatine-kinase enzyme, and the onset of electrocardiogram anomalies.

Complete revascularization was defined when each of three major vascular territories subtended by a significant coronary artery stenosis was grafted. Postoperative low-output cardiac syndrome was defined by a cardiac index value <2.0 L per min per m^2^, requiring the inotropic support for a period greater than 24 h or the use of intra-aortic balloon pump. Preoperative and at weaning form cardiopulmonary bypass hematocrit and hemoglobin levels were evaluated.

Major non-cardiac complications were also analyzed: a pulmonary complication was defined as an episode of primary respiratory failure requiring mechanical ventilation for >48 h, re-intubation, or intermittent application of non-invasive positive-pressure ventilation; permanent neurological complication due to focal or general cerebral lesion was defined as a stroke; transient ischemic attack was defined when neurological symptoms lasted <24 h before disappearing; acute kidney injury was defined as a twofold increase of preoperative serum creatinine level or oliguria requiring need of continuous veno-venous hemodiafiltration.

Operative mortality included death for any causes in-hospital after operation, at anytime or within 30 days after discharge.

### Surgical procedure

CABG surgery was carried out in all patients by complete longitudinal sternotomy, normothermic, or in mild hypothermia cardiopulmonary bypass with right atrial or bi-caval cannulation and arterial cannulation in the ascending aorta, and aortic cross-clamping.

Type of cardioplegia was given in accordance with the surgeons’ choice: one surgeon (G.R.) used warm blood cardioplegia from January 2015 to April 2016, and subsequently crystalloid cold St. Thomas cardioplegia; all the other surgeons employed warm blood cardioplegia throughout the study period.

In the CCC group, intermittent antegrade cold (4 °C) crystalloid cardioplegia (10 mL/kg the first dose, followed by doses of 5 mL/Kg) was administered every 25–30 min. In the WBC group, intermittent antegrade warm (34–35 °C) blood cardioplegia (600 mL the first dose, followed by doses of 400 mL, each in 2 min) was administered every 16–20 min.

Coronary artery bypass surgery was performed using in all cases the left internal mammary artery graft to the left anterior descending artery, in association with saphenous vein grafts, single or in Y-graft composition, to the right coronary artery and/or to the left circumflex artery branches.

### Statistical analysis

Continues variables in tables and text were expressed as mean values plus minus the SD, categorical variables as number and percentage. Statistical analysis was performed with Stat View 4.5 (SAS Institute Inc., Abacus Concepts, Berkeley, CA). The analysis of variance test was used to calculate repeated measures of myocardial enzymes levels at time 0, 24, and 48 h after CABG. The differences between the two groups of patients were calculated by means of the Student’s *t* test for continuous data and the *χ*^2^ or Fisher’s exact test for categorical data. All *P* values <0.05 were considered statistically significant.

## Results

Preoperative characteristics of the two groups are presented in Table 1. Both groups were similar for the preoperative characteristics, except for the clearance of creatinine, that was lower in WBC group (*P* = 0.045). Intraoperative data are summarized in Table 2. Aortic cross-clamp and cardiopulmonary bypass times were similar in both groups. As expected, as compared with CCC group, the mean number of cardioplegia’s doses per patient was higher in WBC group (2.0 ± 0.7 versus 2.3 ± 0.8; *P* = 0.045), despite the mean number of distal coronary artery anastomoses per patient was lower in WBC group (2.7 ± 0.8 versus 3.2 ± 0.9; *P* = 0.001). However, complete revascularization was achieved in all patients of both groups.

**Table 1:** Preoperative characteristics

Characteristics	WBC group (*n*=297)	CCC group (*n*=33)	*P* value
Age, years	67.4 ± 8.9	65.7 ± 9.5	0.295
EuroSCORE II, %	2.52 ± 1.93	2.18 ± 1.69	0.325
Male, *n* (%)	243 (81.8)	29 (87.8)	0.386
Body surface area, m²	1.9 ± 0.2	1.9 ± 0.2	0.568
Body mass index, kg/m²	26.9 ± 3.9	27.8 ± 3.7	0.170
Hypertension, *n* (%)	256 (86.2)	30 (90.9)	0.450
Diabetes on insulin, *n* (%)	63 (21.2)	6 (18.2)	0.685
Chronic lung disease, *n* (%)	20 (6.7)	1 (3.0)	0.408
Extracardiac arteriopathy, *n* (%)	46 (15.5)	4 (12.1)	0.609
Clearance of creatinine (mL per min)	77.3 ± 27.9	88.8 ± 51.0	0.045
Renal impairment on dialysis, *n* (%)	6 (2.0)	2 (6.1)	0.152
NYHA III–IV class, *n* (%)	62 (20.9)	5 (15.2)	0.438
Unstable angina, *n* (%)	143 (48.1)	17 (51.5)	0.714
Recent myocardial infarction, *n* (%)	100 (33.7)	12 (36.4)	0.757
LVEF, %	52.8 ± 8.7	52.4 ± 11.0	0.798
*No. of diseased vessels,* *n* *(%)*			
One	11 (3.7)	1 (3.0)	0.845
Two	61 (20.5)	6 (18.2)	0.919
Three	221 (74.4)	26 (78.8)	0.582
Left main stem	106 (35.7)	12 (36.4)	0.939

*LVEF* left ventricular ejection fraction, *NYHA* New York Heart Association class, *WBC* warm blood cardioplegia, *CCC* crystalloid cold cardioplegia

**Table 2:** Operative variables

Variables	WBC group (*n* = 297)	CCC group (*n* = 33)	*P* value
CPB time, min	97.8 ± 32.5	104.8 ± 36.9	0.243
Cross-clamp time, min	57.2 ± 20.9	63.8 ± 22.6	0.089
No. of cardioplegia’s doses per patient	2.3 ± 0.8	2.0 ± 0.7	0.045
No. of grafts per patient	2.7 ± 0.9	3.2 ± 0.9	0.001
Complete revascularization, *n* (%)	297 (100)	33 (100)	—
Hemoglobin pre-CPB, g/dL	12.6 ± 1.6	12.6 ± 2.2	0.523
Hemoglobin post-CPB, g/dL	9.5 ± 1.2	9.4 ± 1.2	0.759
Hematocrit pre-CPB, %	37.6 ± 4.8	38.1 ± 6.5	0.457
Hematocrit post-CPB, %	28.4 ± 3.4	28.0 ± 3.4	0.637
Intraoperative deaths, *n* (%)	1 (0.3%)	0	0.739

*CPB* cardiopulmonary bypass, *WBC* warm blood cardioplegia, *CCC* crystalloid cold cardioplegia

Before and at the weaning from cardiopulmonary bypass hematocrit and hemoglobin levels were similar in both groups (*P* = not significant, for all comparisons). Postoperative results are reported in Table 3.

**Table 3:** Postoperative results

Variables	WBC group (*n* = 297)	CCC group (*n* = 33)	*P* value
No. of blood units transfused per patient	0.6 ± 1.4	0.2 ± 0.6	0.091
Low cardiac output syndrome, *n* (%)	13 (4.4)	1 (3.0)	0.713
Intra-aortic balloon pump, *n* (%)	1 (0.3)	1 (3.0)	0.059
Pulmonary complications, *n* (%)	8 (2.7)	1 (3.0)	0.913
Cerebrovascular accident, *n* (%)	3 (1.0)	0	0.561
Acute kidney injury, *n* (%)	40 (13.5)	3 (9.1)	0.479
Re-exploration for bleeding, *n* (%)	9 (3.0)	1 (3.0)	0.997
Perioperative MI, *n* (%)	13 (4.4)	1 (3.0)	0.716
LVEF, %	53.1 ± 7.2	51.3 ± 8.6	0.198
Atrial fibrillation, *n* (%)	99 (33.3)	6 (18.2)	0.090
Pacemaker implantation, *n* (%)	1 (0.3)	0	0.742
ICU stay, days	4.0 ± 9.1	3.2 ± 2.4	0.614
Postop. in-hospital stay, days	9.3 ± 11.5	10.2 ± 8.2	0.678
Deaths, *n* (%)	6 (2.0)	0	0.410

*MI* myocardial infarction, *LVEF* left ventricular ejection fraction, *ICU* intensive care unit, *WBC* warm blood cardioplegia, *CCC* crystalloid cold cardioplegia

Operative mortality was 1.8% (*n* = 6); it was 2% (*n* = 6) in WBC group, and absent in CCC group (*P* = not significant). Three deaths were due to cardiac causes, one death to mesenteric ischemia, two deaths to septic shock associated with multi-organ failure, respectively.

**Fig. 1:**
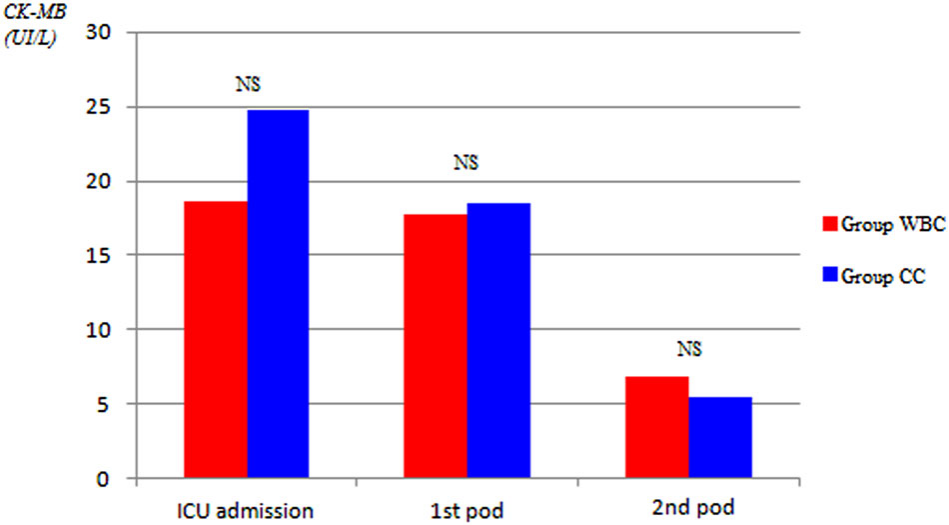
Postoperative release of cardiac isoenzyme of creatine kinase CK-MB. ICU intensive care unit, pod postoperative day

**Fig. 2:**
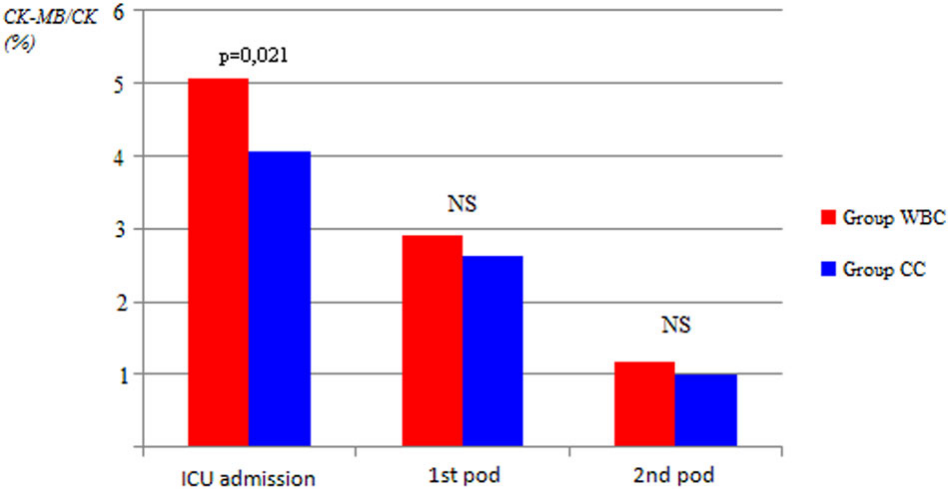
Postoperative ratio >10% between cardiac isoenzyme of creatine kinase CK-MB and total creatine kinase (CK). ICU intensive care unit, pod postoperative day

**Fig. 3:**
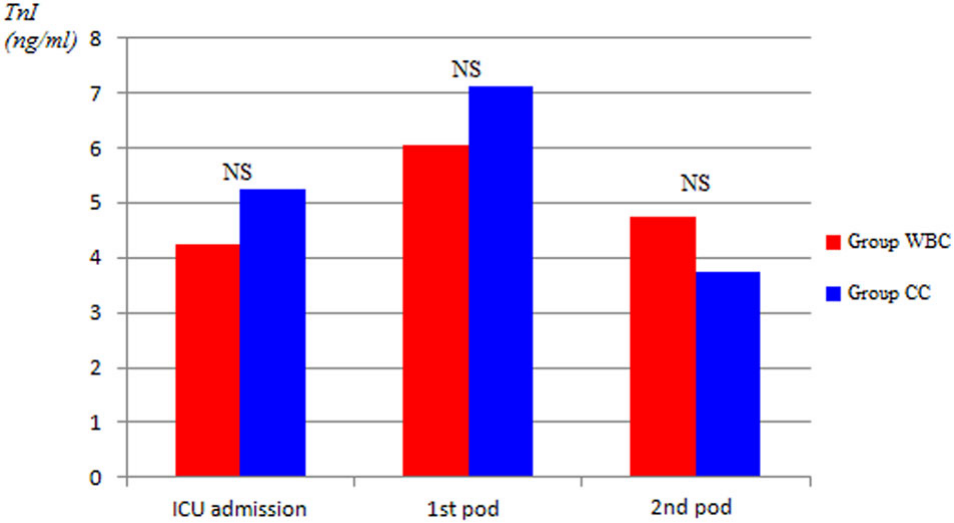
Postoperative release of cardiac troponin I (TnI) . ICU intensive care unit, pod postoperative day

Overall, serum levels of myocardial enzymes (i.e., CK-MB/CK ratio >10% and cardiac troponin I levels, at time 0, 24, and 48 h after CABG operation, respectively) were substantially similar in both groups. In particular, as compared with WBC group, CK-MB/CK ratio >10% was lower during each time points of evaluation in CCC group, with a slight statistical significant difference only at time 0 (4% ± 1.6% versus 5% ± 2.5%) (*P* = 0.021) (Fig. 1 and Fig. 2). Cardiac troponine I release was similar in all times of measurements (Fig. 3).

In both groups, the mean number of blood transfusion units required per patient was similar (Table 3).

In the same way, the incidence of perioperative myocardial infarction, low cardiac output syndrome, stroke, acute kidney injury, pulmonary complications, re-exploration for bleeding, atrio-ventricular blocks requiring need for pacemaker implantation, and the postoperative length of stay were similar in both groups (*P* = not significant, for all comparisons). As compared with CCC group, the incidence of postoperative paroxysmal atrial fibrillation was higher in WBC group (33% versus 18%), although this difference did not reach a statistical significance (*P* = 0.09).

Postoperatively, the mean value of left ventricular ejection fraction was similar in both groups.

## Discussion

Protection method safety during cardiac surgery is determined by the absence of the muscular damage, i.e., impairment of ventricular contraction, and avoidance of increased myocardial enzymes, i.e., perioperative myocardial infarction. The improvement of techniques of myocardial preservation has contributed greatly to significant advances in cardiac surgery. However, several questions remain opened regarding the use of warm versus cold cardioplegia, blood versus crystalloid cardioplegia, antegrade versus retrograde delivery, and intermittent versus continuous perfusion^6, 7^.

The debate over the optimal temperature of cardioplegia during cardiac surgery has been one of the most important aspects of myocardial protection. Early cardioplegic techniques used cold crystalloid solutions to initiate and maintain cardiac arrest during heart surgery, and it remained as a cornerstone of cardiac surgical practice since its introduction in the early 1950s. Although it could lower myocardial oxygen demands and consequently the risk of ischemic damage, cold cardioplegia might inhibit myocardial enzymes and it may result in the delay in metabolic and functional cardiac recovery after surgery. In the hope of maximizing intra-operative myocardial protection, warm blood cardioplegia was first introduced in 1970s^8^. Intermittent perfusions of warm blood cardioplegia were introduced in 1980s and proved to provide excellent myocardial protection during heart surgery^9, 10^.

Numerous randomized controlled trials have been conducted to compare warm cardioplegia with cold cardioplegia for myocardial protection, but the outcomes of these studies remain inconclusive^11–13^.

Fan et al.^14^ in a meta-analysis identifying 41 randomized controlled trials including 5897 patients, compared warm cardioplegia with cold cardioplegia for myocardial protection in patients undergoing heart surgery. The risk of in-hospital death and myocardial infarction was similar in both groups.

Low-output cardiac syndrome caused by cardiac damage from inadequate myocardial preservation is a strong predictor of both perioperative and late death, and it could also prolong hospital stay and costs. The Warm Heart Trial^15^ reported that fewer incidence of postoperative low-output syndrome occurred in the warm cardioplegia group; however, the results from previous mentioned meta-analysis did not show statistical difference between these two types of cardioplegia^14^. Moreover, in the same way, the incidence of stroke and postoperative atrial fibrillation was similar^14^. The main differences of results emerging from the meta-analysis was the reduction in postoperative CK-MB and cardiac troponin concentration with the use of warm cardioplegia as compared with cold.

In another meta-analysis, Guru et al.^16^ analyzed data from 4316 patients who underwent cardiac surgery procedures using blood (cold, tepid, or warm) or cold crystalloid cardioplegia. In their analysis, blood cardioplegia provided superior myocardial protection as compared with crystalloid cardioplegia in terms of reduced incidence of low-output syndrome and reduced CK-MB release, although the incidence of death, myocardial infarction, and low cardiac output syndrome were found to be similar.

In two more recently published meta-analyses performed by Abah et al.^17^ and by Zeng et al.^18^, respectively, on 5897 and 2866 patients undergoing cardiac surgery, the conclusions were different: in the first one, warm and cold cardioplegia resulted in similar short-term mortality and clinical outcomes, in the last one, cold blood cardioplegia reduced the incidence of perioperative myocardial infarction in comparison with cold crystalloid cardioplegia. Kaul et al.^19^ on 123 patients undergoing combined valve (aortic or mitral) and coronary artery bypass surgery found a significant reduced release in AST enzyme in favor of cold blood cardioplegia in comparison with cold crystalloid or with the use of ischemic cardiac arrest. Ascione et al.^20^ found a significant reduced release of cardiac troponine I at 1, 24, 48 h postoperatively in favor of cold blood cardioplegia in comparison with warm blood cardioplegia.

The main limitations of all the reported studies were related to the fact that the patient populations were heterogeneous, as they underwent different procedures of cardiac surgery and not isolated CABG, and that the analyzes were carried out at the same time with the use of cardioplegia warm or cold, blood, or crystalloid, administered intermittently or continuously, retrograde or antegrade^14–18, 21^.

Fiore et al.^22^ in a single-center study showed that intermittent tepid blood cardioplegia was a more efficacious method of myocardial protection as compared with intermittent cold blood cardioplegia in 52 elective CABG patients, in terms of postoperative reduced CK-MB enzyme release (*P* < 0.04), improved left ventricular function (*P* < 0.05), and decreased need for inotropic support (*P* < 0.05), although, as reported, with small statistical differences.

Analysis of our results has been focused on some main aspects, clinical and surgical. The clinical bottom line is that warm blood or cold crystalloid cardioplegia resulted in similar short-term mortality and rate of postoperative complications after isolated CABG. The slight statistical difference in the value of CK-MB/CK ratio >10% at the admission in intensive care unit, at time 0 (Fig. 1), did not translate into different clinical outcomes in the two groups of patients, thus showing both types of cardioplegia as an equivalent efficacious method of myocardial protection during cardiac arrest.

From a surgical point of view, crystalloid cold cardioplegia, which may be repeated every 25–30 min, may offer some advantage by allowing a longer time available for the packaging of distal coronary artery anastomoses and an operating bloodless filed at the site of the anastomosis. Moreover, there was not a significant difference in the value of hemodilution at the weaning from cardiopulmonary bypass in comparison with the administration of blood cardioplegia. On the contrary, if the surgeon prefers to use the warm cardioplegia, must pay more attention to the time of administration, being required a shorter time of repetition of the dose to achieve an effective myocardial protection during cardiac arrest.

This study has several limitations. It is a retrospective and observational analysis: for this reasons, inherent limitations are present. Moreover, the analyzed sample of the CCC group is quite small, although this study takes into account a short surgical period in order to avoid bias, and the population in question regarded isolated coronary artery surgery and appeared to be homogeneous.

## Conclusions

We have observed that in the presence of a complete revascularization of coronary territories, in-hospital results were not affected by the use of two different types of antegrade intermittent cardioplegia frequently used in the clinical practice to achieve the cardiac arrest during on-pump CABG. Both strategies appear to allow an equivalent and a satisfactory method for myocardial protection during the period of cardiac arrest. As compared with cold crystalloid, warm blood cardioplegia requires a shorter interval of administration to achieve better myocardial protection. Therefore, the choice of one type of cardioplegia respect to each other remains at discretion of the surgeon.
